# Slowing myopia progression with cylindrical annular refractive elements (CARE) spectacle lenses—Year 1 results from a 2‐year prospective, multi‐centre trial

**DOI:** 10.1111/aos.16795

**Published:** 2024-11-15

**Authors:** Xiaoqin Chen, Min Wu, Cui Yu, Arne Ohlendorf, Katharina Rifai, Christina Boeck‐Maier, Siegfried Wahl, Youhua Yang, Yi Zhu, Lihua Li, Padmaja Sankaridurg

**Affiliations:** ^1^ Tianjin Eye Hospital, Tianjin Key Lab of Ophthalmology and Visual Science Tianjin People's Republic of China; ^2^ Tianjin Eye Hospital Optometric Center Tianjin People's Republic of China; ^3^ Beijing Tongren Vision Care, Beijing Tongren Hospital Capital Medical University Beijing People's Republic of China; ^4^ He Eye Specialist Hospital Shenyang Liaoning People's Republic of China; ^5^ ZEISS Vision Care, Aalen, Germany, Carl Zeiss Vision International GmbH Aalen Germany; ^6^ Institute for Ophthalmic Research Eberhard Karls Universitat Tubingen Tuebingen Baden‐Wuerttemberg Germany; ^7^ ZEISS Vision Care, Carl Zeiss Vision (Guangzhou) Ltd. Guangzhou People's Republic of China; ^8^ School of Optometry and Vision Science, Faculty of Medicine and Health University of New South Wales Sydney New South Wales Australia

**Keywords:** axial length, myopia control, spectacle lenses

## Abstract

**Purpose:**

To evaluate the effectiveness of 12 months of spectacle lens wear incorporating cylindrical annular refractive elements (CARE) in slowing myopia progression compared to single vision (SV) spectacle wear.

**Methods:**

In an ongoing 2‐year prospective, double‐masked, multi‐centre clinical trial, 240 Chinese children aged 6–13 years, spherical equivalent refractive error (SE) −0.75 D to −5.00 D were randomised to one of three groups of 80 participants each to wear: SV spectacle lens (*N* = 80), CARE spectacles (7 mm central clear zone surrounded by treatment zone incorporating CARE with mean surface power of +4.6 D) and CARE S (9 mm central clear zone surrounded by treatment zone comprising CARE with mean surface power of +3.8 D). Cycloplegic SE and axial length (AL) were measured at 6‐month intervals.

**Results:**

Compared to baseline, changes in SE and AL were significantly different between the groups at both 6 and 12 months (*p* < 0.001, linear mixed model). Adjusting for site, group, parental myopia and age, at 12 months, the estimated change in SE and AL with 95% CI with SV was −0.65 D (CI: −0.56 to −0.74 D)/0.32 mm (CI: 0.29–0.36 mm). In comparison, the estimated change in SE/AL at 12 months with CARE was −0.35 D (−0.26 to −0.44 D)/0.19 mm (0.15–0.22 mm) and with CARE S was −0.36 D (−0.27 to −0.46 D)/0.21 mm (0.18–0.25 mm) at 12 months. Progression was slower with CARE and CARE S compared to SV (*p* < 0.05) but did not differ from each other (*p* = 0.793 and 0.336 for SE and AL, respectively).

**Conclusions:**

In children with myopia, after 12 months of lens wear, both CARE and CARE S spectacle lenses significantly slowed myopia progression compared to SV lenses.

## 1 INTRODUCTION

While myopia is more prevalent in East Asian and South‐East Asian countries, growing evidence indicates a global increase in the prevalence of myopia. However, the consequences of myopia extend beyond (Sankaridurg et al., 2021). The condition results in an economic burden to the individual and the society and also includes loss of productivity due to uncorrected or undercorrected myopia. Additionally, it may lower the quality of life, and higher levels of myopia are associated with an increased risk of developing sight‐threatening complications (Haarman et al., 2020). Importantly, evidence indicates that in recent years, myopia has been occurring at younger ages than before (Ma et al., 2016). Since the progression of myopia is greater at younger ages, those with an early onset are likely to reach higher levels of myopia placing them at a greater risk of developing complications as well as increasing the overall burden (Chiang et al., 2021; Chua et al., 2016; Donovan et al., 2012; Sankaridurg & Holden, 2014). Therefore, it is necessary to slow or minimise the progression of myopia in those who are already myopic.

Over the past two decades, many optical, pharmaceutical, light‐based and combination strategies were developed to slow the progression of myopia (Jong et al., 2021; Sankaridurg et al., 2023; Wildsoet et al., 2019). These strategies, especially optical strategies stemmed mostly from data generated from animal models that demonstrated convincingly that ocular growth is modulated by visual feedback (Baird et al., 2020; Troilo et al., 2019). Meta‐analyses indicate heterogeneity in the effectiveness of various myopia control strategies, however, many of these strategies with the exception of undercorrection and rigid gas permeable contact lenses were found to slow myopia (Lawrenson et al., 2023; Youssef et al., 2024). Of the optical approaches, spectacle‐based strategies are considered to be a more convenient and safer option especially for young children with myopia. Furthermore, with modelling of costs and cost‐effective solutions for a lifetime of myopia, spectacle‐based strategies feature as an economical option (Fricke et al., 2023).

Although the evidence for visual feedback and ocular growth is confirmed, the specific pathways and mechanisms underlying onset and progression remain unclear. Hyperopic defocus at either central and/or peripheral retina is thought to play a role in axial elongation and strategies that employ relative positive power over specific areas of the lens to induce myopic defocus across the retina found success in slowing myopia. In animal experiments, dual focus lenses that incorporated concentric annular powers that were relatively more positive than the central power were found to consistently slow eye growth (Smith III et al., 2020).

In the current trial, we evaluated the use of a spectacle‐based strategy that incorporated annular elements that were cylindrical in shape and imposed myopic defocus at one of the image planes. The objective was to determine if use of such cylindrical refractive elements is successful in slowing the progression of myopia in children with myopia.

## 2 MATERIALS AND METHODS

### 2.1 Patient population

In an ongoing 2‐year prospective, parallel‐group, double‐masked, clinical trial (NCT 05288335), 240 Chinese children with myopia were enrolled and randomised to one of three parallel arms in the trial. The study is a multi‐centre trial conducted at three sites across China: Tianjin Eye Hospital Optometric Center, in Tianjin (Site 1), Shenyang He Eye Hospital in Shenyang (Site 2) and Beijing Tongren Hospital in Beijing (Site 3). At enrolment, all participants were aged 6–13 years, had refractive error ranging from −0.75 D to −5.00 with astigmatism ≤1.50 D, anisometropia of ≤1.50 D, had best corrected visual acuity of ≥1.0 in both eyes and had parents/carers willing to sign the informed consent. Children with any pre‐existing ocular or systemic conditions or diseases, those with any history of ocular trauma or intraocular surgery, any significant slit lamp findings that would impact lens wear, those with intraocular pressure <10 mm Hg or >21 mm Hg, fundus findings or grade 2 or greater, current use of myopia control products or participation in a clinical trial of a drug (e.g. atropine) within the past 3 months or those that could not commit to frequent visits were excluded from the trial. The study was approved by the Medical Research Ethics Committee of Tianjin Eye Hospital (KY202110), Shenyang He Eye Hospital Clinical Trials Organisation and Medical Ethics Committee (IRB(2021)K015.01) and the Medical Ethics Committee of Peking Tongren Hospital, Beijing (TRECKY2021‐235). Study procedures adhered to the Declaration of Helsinki for Experimentation on human subjects. Parents and/or carers of the participants provided the informed consent.

### 2.2 Study design and investigational lenses

**FIGURE 1:**
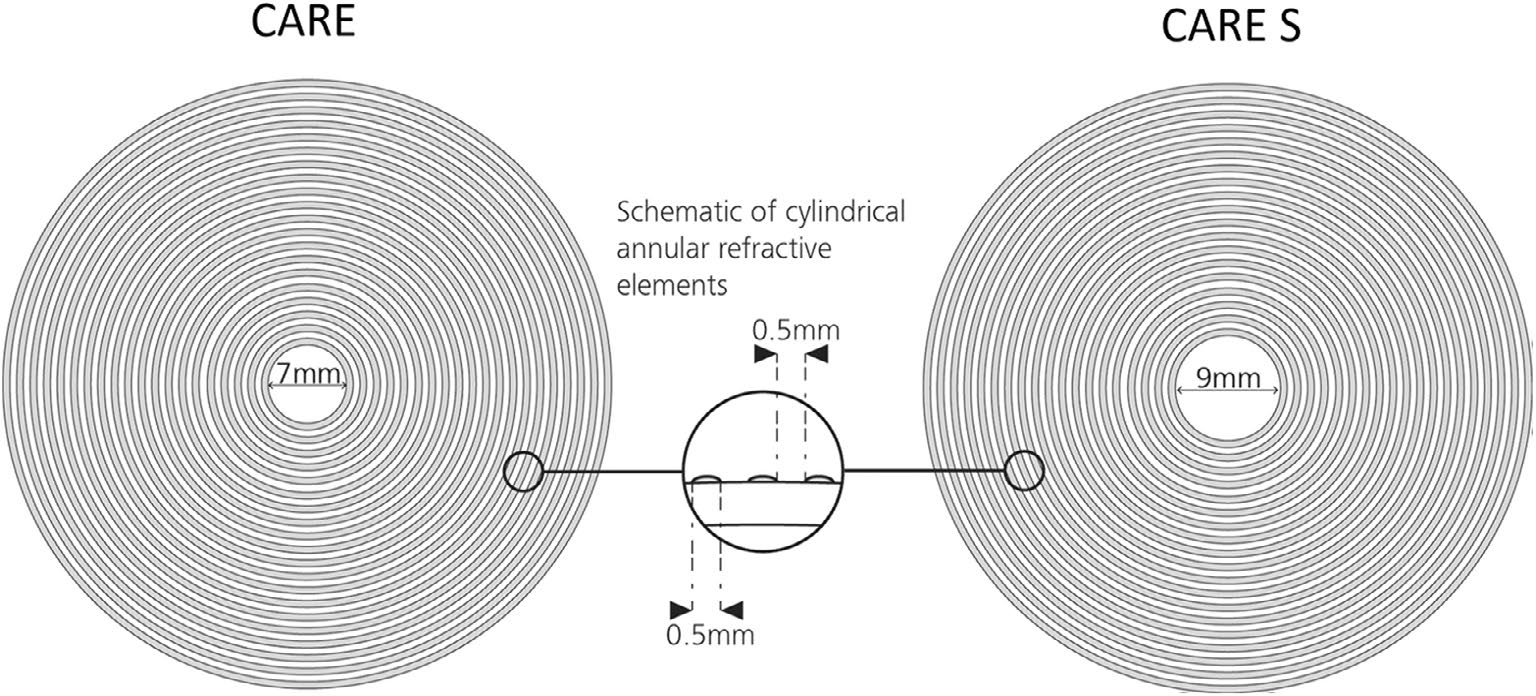
Schematic illustration of the features of CARE and CARE S spectacle lenses.

Each of the sites enrolled a specific number of individuals randomised to one of three groups in a block randomisation: two test lenses or a control lens on a ratio of 1:1:1. Randomisation for the study was generated using means of block randomisation in SAS software via a randomisation list. All study visits took place at one of the three trial sites. Both test and control spectacle lenses were manufactured by ZEISS Vision Care International GmbH (Germany). Both the test and control spectacle lenses were made of an MR8 (Mitsui Resin, refractive index 1.60) lens material and incorporated a hard coating and a multi‐layer anti‐reflection coating. The control spectacle lens was a standard single‐vision spectacle lens. Two test lenses were used in the trial. The CARE lens designs comprise a central aperture that corrects for the distance refractive error of the eye. Surrounding this central zone is a treatment zone that included concentric cylindrical elements. The cylindrical elements are arranged in concentric rings and have a convex curvature in one meridian that results in relatively more positive power compared to the base surface power in one image plane. The cylindrical elements are alternated between the clear zones that correct for the distance refractive error of the eye. Due to the distinct geometry of the cylindrical elements, light passing through the elements does not refract to a single point, but in combination with the adjacent elements and alternating clear zones, it instead creates a blended distribution of myopic defocus in front of the retina. Furthermore, the test lenses feature a back surface design that is aspherical and with less minus power towards the periphery of the lens to minimise peripheral retinal hyperopic defocus. The two test lenses differ in the size of the central zone and the mean surface power of the cylindrical elements. Test lens 1 (CARE) incorporates a 7 mm central zone, whereas test lens 2 (CARE S) had a 9 mm central zone. The nominal power of the cylindrical elements for test lens 1 (CARE) is +9.2 D and translates to a mean relative positive power of +4.6 D, whereas with test lens 2 (CARE S), the nominal power of the cylindrical elements was +7.6 D and translated to a mean relative positive power of +3.8 D (Figure 1). The two lenses were selected to determine if a higher positive power as with CARE compared to CARE S provides a greater myopia control efficacy.

### 2.3 Study procedures

Once the participant's eligibility was determined, a baseline examination was conducted and children were randomised to one of the three groups to wear either CARE, CARE S or the control single‐vision spectacle lenses. Visual acuity (unaided and best corrected) was determined and cycloplegic autorefraction and axial length (AL) measurements were conducted by a masked examiner. At the end of the visit, frames were chosen and measurements for fitting of the lens wear were taken. At the dispensing visit, visual acuity was measured with the lens in place and thereafter lenses were dispensed. The child and their carer were masked to the lens type. Follow‐up visits were performed at 1 week (phone follow‐up only), 3 and 6 months thereafter. Cycloplegic autorefraction and AL measurements were conducted at 6‐ and 12‐month visits by a masked examiner. A subjective questionnaire addressing various aspects of vision (distance and near vision, vision when going up and down stairs and when walking) with lens wear was completed at baseline, 1 week and 3 months. Cycloplegic autorefraction was conducted using the NIDEKARK‐1 autorefractor and an IOLmaster was used to measure the AL. Eyes were cycloplegic with the use of 1% cyclopentolate instilled as three drops and 10 min apart. Following the instillation of the cycloplegic and approximately 30 min after instillation, the pupils were checked for dilation and responsiveness to light. When cycloplegia was considered to be adequate, measurements were taken. An average of a total of five measurements were considered for autorefraction and an average of five measurements were considered for AL measurements.

### 2.4 Sample size and statistical analysis

Based on the annual progression of myopia from a previously published study (Bao et al., 2022), the target effect size was considered 0.1 mm between the test and control groups. Assuming a standard deviation of 0.2 mm, it was estimated that 64 participants per group would be needed at an alpha of 0.05 and power of 80%. Adjusting the sample size considering a drop out of 20% over 2 years, a total of 80 participants were enrolled in each group. A total of 240 participants were enrolled and randomised to 80 children each to wear of CARE, CARE S and single‐vision spectacle (SV) lenses. All participants were dispensed with the trial lenses and continued in the study. While Sites 1 and 3 enrolled 84 participants each (28 participants for each of the test and control groups, respectively), Site 2 enrolled 72 participants (24 participants for each of the test and control groups, respectively).

Spherical equivalent refractive error (SE) and AL measurements were recorded on an interval scale. SE was computed as sphere + (Cylinder/2). Data from across the three sites is presented as pooled data. For each participant, change from baseline for both SE and AL were determined. Progression in SE and AL were analysed using the intent to treat model. Since there is a high correlation in data between the right and left eyes (AL = 0.95, SE = 0.88), only data from the right eyes are presented. A linear mixed model was conducted after adjusting for relevant confounders that were selected based on the Aikaike information criterion. The model included intervention or lens type, age, gender, parental myopia and compliance. Survival analysis was determined for fast annual progression (≤−0.75 D) using Kaplan–Meier survival analysis with a log‐rank test for differences between groups. Additionally, baseline characteristics of fast progressors versus slow progressors were analysed for each group, and differences were assessed using chi‐squared or ANOVA.

Subjective responses to aspects of lens wear were rated on a scale of 1–4 (1 = poor, 4 = very good) for aspects of lens wear such as distance, intermediate and near vision. At the dispensing visit, participants were asked to rate their vision for distance, near, while walking or doing sport and if they experienced any distortion with their spectacles. In addition to these questions, at 1 week and 1 month, children were asked to rate their comfort and vision when walking the stairs and ease of adaptation. Differences in subjective ratings between groups were analysed using a linear mixed model.

Statistical significance was maintained at *p* < 0.05. Statistical analyses were performed using SAS version 9.4. and MATLAB, Version 9.14.0.2206163 (R2023a) with Statistics and Machine Learning Toolbox Version 12.5, R2023a.

## 3 RESULTS

Table 1 presents the age, SE and AL at baseline for participants enrolled across the three sites and shows that there were no differences between the groups. Table 2 presents the baseline characteristics of the 240 participants randomised to CARE, CARE S and SV groups.

**TABLE 1:** Baseline characteristics and data of subjects across the sites.

Characteristic	Site 1 (*n* = 84)	Site 2 (*n* = 72)	Site 3 (*n* = 84)	*p*‐value
Age (years)	9.9 ± 1.8	10.0 ± 1.7	9.6 ± 1.4	0.200
Gender (male: female)	32 (38.1%): 52 (61.9%)	41 (56.9%): 31 (43.1%)	44 (52.4%): 40 (47.6%)	0.045
Parental myopia none: one: both	19: 26: 30	16:28: 21	18:18:26	0.567
Cycloplegic SE (D)	−2.43 ± 1.06	−2.34 ± 1.12	−2.27 ± 1.12	0.660
AL (mm)	24.63 ± 0.49	24.59 ± 0.68	24.45 ± 0.93	0.240

Abbreviations: AL, axial length; SE, spherical equivalent refractive error.

**TABLE 2:** Baseline characteristics and data of participants across the different groups.

Characteristic	CARE	CARE S	Single vision	*p*‐value
Age (years)	9.9 ± 1.7	9.8 ± 1.7	9.8 ± 1.6	0.215
Age of onset of myopia (years)	9.3 ± 1.7	9.3 ± 1.5	9.2 ± 1.7	0.918
Gender (male: female)	45 (36.6%)/35 (29.9%)	39 (31.7%)/41 (35.0%)	39 (31.7%)/41 (35.0%)	0.549
Parental myopia				
None	14 (20.5%)	21 (31.3%)	18 (26.9%)	0.713
One	26 (38.2%)	23 (34.3%)	23 (34.3%)	
Both	28 (41.2%)	23 (34.3%)	26 (38.8%)	
Cycloplegic SE (D)	−2.23 ± 0.98	−2.30 ± 1.06	−2.31 ± 1.01	0.902
AL (mm)	24.44 ± 0.73	24.34 ± 0.74	24.43 ± 0.73	0.870

Abbreviations: AL, axial length; SE, spherical equivalent refractive error.

### 3.1 Characteristics of participants

**FIGURE 2:**
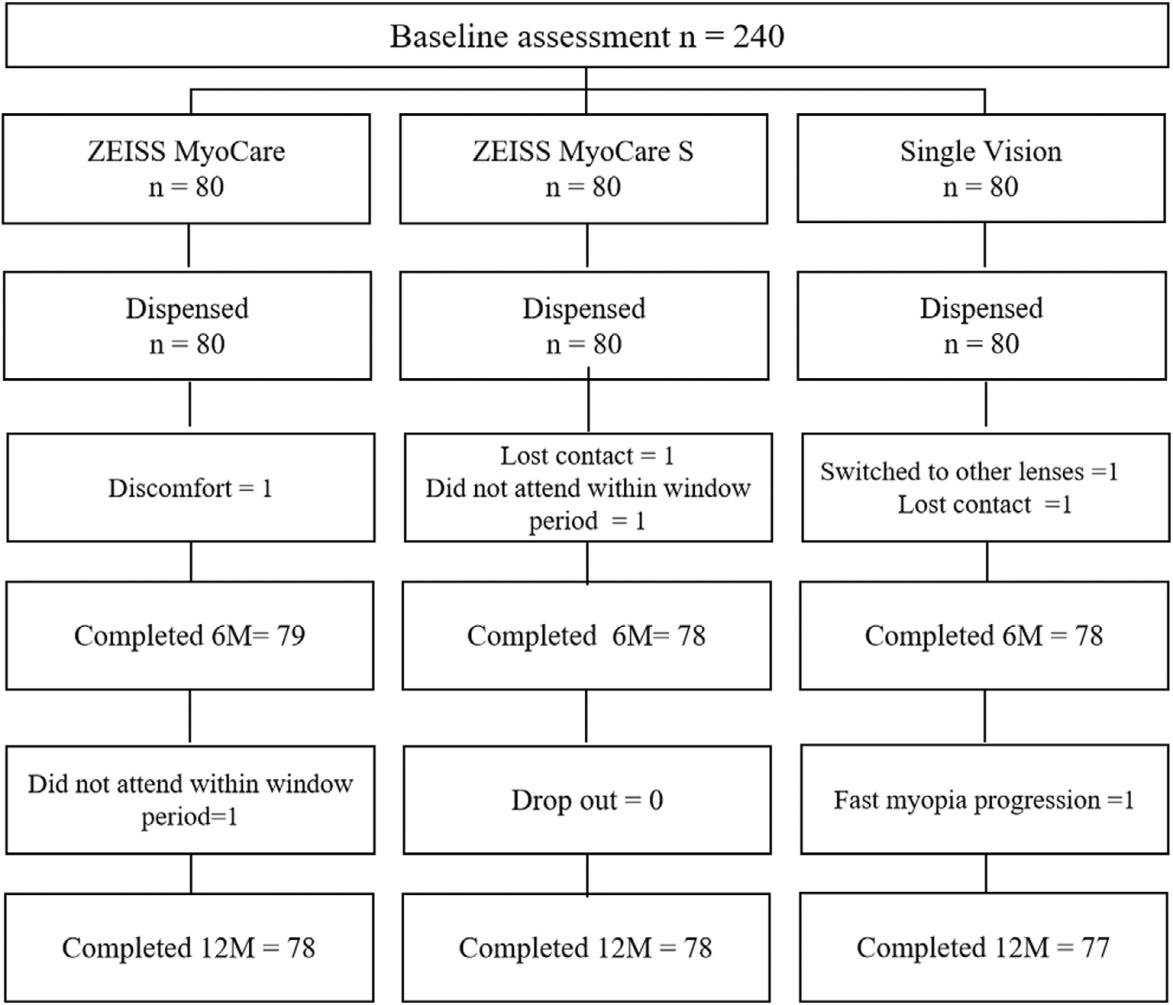
Flow diagram of the participant flow for the 12 months period.

A total of 233 participants (78, 78 and 77 participants in CARE, CARE S and SV groups, respectively) completed 12 months in the trial. The trial flow is presented in Figure 2. Of the two participants who did not complete 12 months in CARE, one participant reported discomfort and the other participant did not return for the study visit within the scheduled period. With CARE S, one of the participants missed attending the visit within the scheduled period and the other participant was lost to follow‐up. Of the three participants who discontinued from SV lens wear, one participant was lost to follow‐up, another participant switched lens type and the third participant reported fast progression. The overall discontinuation rate was 2.5%, 2.5% and 3.75% with CARE, CARE S, and single‐vision lenses, respectively. Baseline characteristics such as age, SE, AL, gender and parental myopia were examined and there were no differences between the groups (Table 2).

### 3.2 Efficacy in slowing myopia progression: Change in SE and AL

After 12 months of lens wear, both CARE and CARE S were observed to exhibit less progression in SE and AL compared to the control group. The observed progression in SE and AL with single‐vision spectacles was −0.29 ± 0.33 D/0.17 ± 0.09 mm at 6 months and −0.65 ± 0.40 D/0.32 ± 0.17 mm at 12 months. In comparison, observed change in SE and AL with CARE and CARE S was slower at −0.10 ± 0.32 D/0.10 ± 0.10 mm and −0.13 ± 0.27 D/0.11 ± 0.11 mm at 6 months (*p* = 0.001 and <0.001, SE and AL, linear mixed model) and −0.37 ± 0.39 D/0.20 ± 0.16 mm and −0.38 ± 0.38 D/0.22 ± 0.15 mm at 12 months (*p* < 0.001, both SE and AL, linear mixed model) respectively.

**FIGURE 3:**
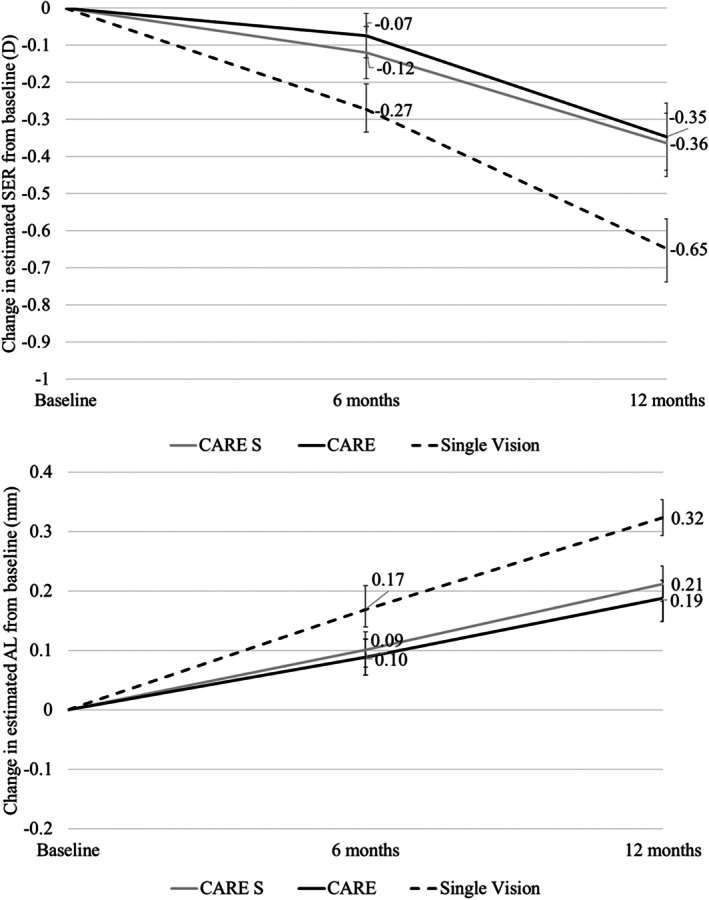
Changes in spherical equivalent refractive error and axial length from baseline to 12 months (error bars are 95% CI).

After adjusting for confounders (age, intervention type, site and parental myopia), the estimated change in SE and AL with single‐vision spectacles was −0.27 D (−0.20 to −0.35 D)/0.17 mm (0.15–0.19 mm) at 6 months and −0.65 D (−0.56 to −0.74 D)/0.32 mm (0.29–0.36 mm) at 12 months. In comparison, changes in SE/AL with CARE were −0.07 D (0.00 to −0.15 D)/0.09 mm (0.07–0.11 mm) at 6 months and −0.35 D (−0.26 to −0.44 D)/0.19 mm (0.15–0.22 mm) at 12 months. With CARE S, changes in SE and AL were −0.12 D (−0.05 to −0.19 D)/0.10 mm (0.08–0.12 mm) at 6 months and −0.36 D (−0.27 to −0.46 D)/0.21 mm (0.18–0.25 mm) at 12 months. Details are estimated of adjusted change in SE and AL are presented in Table 3 and in Figure 3. The absolute values for SE and AL are presented in Table S1.

**TABLE 3:** Intent to treat analysis: observed and adjusted change (baseline to 12 months) in spherical equivalent and axial length with 95% confidence intervals.

Visit	Measure	CARE	CARE S	Single vision
*Observed change*				
6 months	AL (mm)	0.10 ± 0.10	0.11 ± 0.11	0.17 ± 0.09
	SE (D)	−0.10 ± 0.32	−0.13 ± 0.27	−0.29 ± 0.33
12 months	AL (mm)	0.20 ± 0.16	0.22 ± 0.15	0.32 ± 0.17
	SE (D)	−0.37 ± 0.39	−0.38 ± 0.38	−0.65 ± 0.40
*Adjusted change* ^*^.				
6 months	AL (mm)	0.09 (0.07 to 0.11)	0.10 (0.08 to 0.12)	0.17 (0.15 to 0.19)
	SE (D)	−0.07 (−0.15 to 0.00)	−0.12 (−0.19 to −0.05)	−0.27 (−0.35 to −0.20)
12 months	AL (mm)	0.19 (0.15 to 0.22)	0.21 (0.18 to 0.25)	0.32 (0.29 to 0.36)
	SE (D)	−0.35 (−0.26 to −0.44)	−0.36 (−0.27 to −0.46)	−0.65 (−0.56 to −0.74)

Abbreviations: AL, axial length; SE, spherical equivalent refractive error.

* Adjusted for age, site, parental myopia and intervention.

The absolute mean difference in SE and AL at 12 months between the single vision control and CARE lens was 0.31 D and 0.13 mm. Similarly, the absolute mean difference in SE and AL at 12 months between the control group and CARE S was 0.29 D and 0.11 mm. Post hoc analysis indicated that both test lenses were different from control (*p* < 0.05), however, there was no difference between the two test groups (*p* = 0.394 and 0.793 for SE and *p* = 0.443 and 0.336 for AL at 6 and 12 months, respectively).

### 3.3 Probability of survival of fast progression (0.75 D or more) over 12 months

**FIGURE 4:**
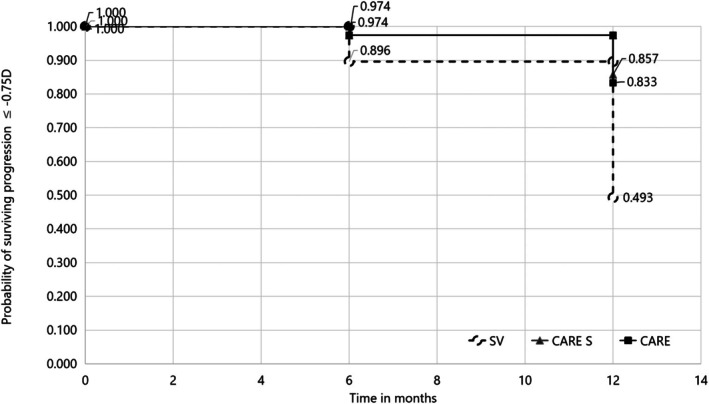
Probability of surviving fast progression, that is, −0.75 D or worse.

Figure 4 presents the survival analysis for eyes that survived fast progression (≥0.75 D). At both 6 and 12 months, both CARE and CARE S had a better chance of surviving progression of ≥0.75 D. In the SV group, of the 77 wearers, 11 (11.6%) s and 39 (51.7%) had progression of ≥0.75 D at 6 and 12 months, respectively. In comparison, only 2 (2.6%) and 11 (16.7%) of CARE wearing eyes and only 2 (2.6%) and 11 (14.3%) of CARE S‐wearing eyes had progression of ≥0.75 D at 6 and 12 months. While the differences in survival between SV and CARE groups were significant, the survival of CARE groups was not different to each other (CARE vs. SV *p* < 0.0001, CARE S vs. SV *p* < 0.0001, CARE vs. CARE S *p* = 0.69).

Table 4 presents the baseline characteristics of fast progressors (≥0.75 D) versus slow progressors across the three groups. Across all groups, fast progressors were younger than slow progressors. Although there were differences between fast and slow progressors for baseline SE and AL, there was no consistent pattern between groups.

**TABLE 4:** Characteristics of fast (−0.75 D or worse) and slow (<−0.75D) progressors.

Characteristic	CARE			CARE S			Single vision		
	Fast progressors (*n* = 11)	Slow progressors (*n* = 67)	*p*‐value	Fast progressors (*n* = 11)	Slow progressors (*n* = 67)	*p*‐value	Fast progressors (*n* = 39)	Slow progressors (*n* = 38)	*p*‐value
Age	8.5 ± 1.2	9.9 ± 1.6	0.004	9.4 ± 1.6	10.1 ± 1.7	0.052	9.6 ± 1.8	10.1 ± 1.3	0.036
Gender (male: female)	63.6: 36.4	44.7: 55.3	0.246	45.4: 54.6	56.7: 43.2	0.486	63.1: 36.8	38.4: 61.5	0.030
Parental myopia none: one: two	25.0: 12.5: 62.5	31.0: 37.9: 31.0	0.184	12.5: 25.0: 62.5	22.4: 39.6: 37.9	0.414	19.4: 38.7: 41.9	35.2: 29.4: 35.2	0.352
Baseline SE (D)	−2.78 ± 1.32	−2.22 ± 1.01	<0.001	−2.11 ± 1.02	−2.24 ± 0.95	<0.001	−2.28 ± 1.00	−2.31 ± 1.04	<0.001
Baseline AL (mm)	24.46 ± 1.04	24.32 ± 0.70	<0.001	24.26 ± 1.04	24.49 ± 0.66	<0.001	24.51 ± 0.81	24.38 ± 0.66	<0.001

Abbreviations: AL, axial length; SE, spherical equivalent refractive error.

### 3.4 Compliance and subjective impressions to lens wear

**FIGURE 5:**
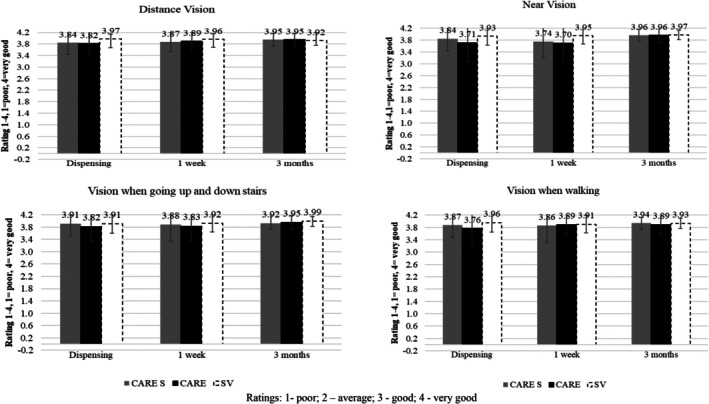
Subjective impressions of visual performance across the lens groups (scale of 1–4, where 1 = poor, 2 = average, 3 = good, 4 = very good). Error bars are S.D.

The average wearing time was high for all groups with an average wearing time of 13.5 ± 1.4, 13.3 ± 1.2 and 13.1 ± 2.0 h/day for CARE, CARE S, and SV, respectively, with no differences between the groups (*p* = 0.18). Across all groups, only 2% reported spectacle wear of <12 h/day. For all aspects of lens wear, subjective ratings for vision for distance, near, when going up and down stairs and when walking was high and >3.5 (scale of 1–4 where 1 = poor, 4 = very good) (Figure 5). After 1 week of wear, there were no differences between the groups in their respective ratings for distance vision, vision when walking stairs and perception of moving objects. Near vision was rated lower with CARE and CARE S at 1 week compared to SV (3.70 ± 0.61 and 3.74 ± 0.55 vs. 3.95 ± 0.28, *p* = 0.04 and *p* = 0.01) but improved to SV level at 3 months (3.96 ± 0.26 and 3.96 ± 0.19 vs. 3.97 ± 0.16).

## 4 DISCUSSION

The 12‐month interim analysis of this ongoing, randomised, double‐masked prospective trial found that when compared to SV lenses, both CARE and CARE S spectacle lenses that incorporated cylindrical annular refractive elements slowed progression of myopia. With both the test lenses, the difference in the 12‐month progression, both with respect to change in spherical equivalent refractive error and axial length as compared to single‐vision lenses was significant at both 6 and 12 months. At 12 months, after adjusting for confounders, the difference in progression with CARE was 0.31 D and 0.13 mm and with CARE S was 0.29 D and 0.11 mm. In a network meta‐analysis, the estimates of effect with peripheral plus spectacle lenses at 1 year were 0.28 D less change in spherical equivalent and 0.14 mm less change in axial length as compared to controls (Lawrenson et al., 2023). The resultant difference of >0.25 D and more than 0.10 mm difference in axial elongation with CARE lenses after 1 year corresponded to the effect size reported with peripheral plus spectacles by Lawrenson et al., 2023 and considered meaningful in terms of clinically important differences (Lawrenson et al., 2023). Moreover, the risk of fast progression (−0.75 D or more) was significantly reduced with CARE lenses compared to single‐vision lenses. Over the 12‐month period in lens wear, discontinuations from spectacle wear were very few at 2.5%, 2.5% and 3.75% with CARE, CARE S and SV lenses, respectively. Thus, the technology used in this trial, with the incorporation of cylindrical annular elements imposing myopic defocus at the retina, offers an effective way of slowing the progression of myopia while maintaining adequate visual performance.

Although relative per cent efficacy is commonly employed (Wildsoet et al., 2019), it was suggested that absolute reduction in AL and/or SE should be considered the relative efficacy metric may be influenced by factors such as the duration of the trial and the progression rate of the control group (Brennan et al., 2021). Considering the results from other recent randomised clinical trials that compared the progression of spectacle and contact lenses designed to slow myopia over a 1‐year period, the absolute difference in AL compared to control group ranged from 0.23 to 0.03 mm and was 0.53–0.08 D for SE (Bao et al., 2022; Chamberlain et al., 2019; Chen et al., 2024; Lam et al., 2022; Liu et al., 2023, 2024; Paune et al., 2015; Rappon et al., 2023; Sankaridurg et al., 2019; Walline et al., 2020; Yuval et al., 2024). Given these values, both the test lenses performed favourably with an absolute difference of 0.13 and 0.11 mm for AL and 0.31 D and 0.29 D with CARE and CARE S, respectively (Lawrenson et al., 2023). However, it should be noted that diminishing efficacy has been reported for subsequent years in treatment (Sankaridurg et al., 2023; Wolffsohn et al., 2019) and long term efficacy of CARE lenses remains to be determined.

More importantly, in a real‐world study conducted in China that evaluated two myopia control spectacles (Stellest and DIMS) as well as two orthokeratology treatments, the 1‐year axial elongation in eyes (unadjusted) using the treatments ranged from 0.19 to 0.20 mm (Yang et al., 2024). Unadjusted axial elongation observed with CARE and CARE S at 0.20 and 0.22 mm indicates similar performance with little difference in axial elongation across the various myopia control approaches.

The chance of surviving fast progression (−0.75 D or more over 1 year) was determined and was found to be greater with CARE lenses. Nearly one of every two eyes wearing SV lenses experienced fast progression (50.7%). In comparison, only 16.7% and 14.3% of CARE and CARE S wearers experienced fast progression. Across all groups, fast progressors were slightly younger but there were no other significant features that would identify fast progressors.

In a previous trial that also utilised cylindrical annular elements, a difference of only 0.14 D and 0.09 mm was reported (Liu et al., 2023), the previous lens had a much larger central aperture of 9.4 mm and also differed in the power of the cylindrical elements and the interannular spacing. It is not clear if any of these differences contributed to the variance in the study results. Furthermore, unlike the current trial and many other previous trials, where greater differences between test and control groups are commonly observed early during lens wear, in the trial conducted by Liu et al., there was little difference in the test and control lenses during the first 6‐month period of the trial but an improved efficacy observed during the second 6‐month period (Liu et al., 2023). It was reported that this may be related to better patient adherence. Interestingly, in some of the previous trials, high compliance was observed to improve efficacy (Lam et al., 2014; Sankaridurg et al., 2019). However, given the high level of compliance in the current trial (mean wearing time of 13.3 h), we were unable to assess the effect of compliance on efficacy.

With the cylindrical annular elements, light directed through the elements results in one of the focal planes in myopic defocus at the retina. Combined with the alternating zones that correct for the distance refractive error or the distance optic, they result in overlapping areas that result in an elongated zone of myopic defocus in front of the retina. Both the test lenses were effective in slowing progression of myopia; although CARE has slightly better efficacy, the differences were not significant. In another study that considered two spectacle lens designs with varying amounts of defocus delivered at the retina through aspherical lenslets on the lens, the lens design with a higher amount of defocus was found to result in greater efficacy (Bao et al., 2022). No such differences were seen in this trial with the two test lenses and therefore, considering efficacy alone, both lenses would be equally suitable for any individual with myopia. This needs to be confirmed in future follow‐ups. However, reviewing the subjective assessments from the current trial, although not significant, at dispensing, slightly greater ratings were observed for CARE S than CARE. This may be a benefit of a larger clear central zone devoid of the cylindrical elements and a lower magnitude of positive power with the cylindrical annular elements. The results indicate that, therefore, it may be possible to increase the clear zones on the lens to facilitate vision and adaptation without sacrificing efficacy. It is possible that if there are any issues with adaptation, CARE S might be a suitable strategy. Furthermore, in a trial involving DIMS lenses, adult wearers were less tolerant of the visual experience provided by the lenses compared to children (Lu et al., 2020). Therefore, in older children where progression is slower (Sankaridurg & Holden, 2014), CARE S might be a more suitable strategy.

The change in SE and AL in the control SV group was −0.65 ± 0.33D/0.32 ± 0.17 mm. The adjusted mean change in SE and AL for a mean age of 9.8 years at baseline was −0.65 D and 0.33 mm. Given the difficulty with enrolling children into prospective clinical trials, it was suggested that virtual controls be used to estimate change in progression. The estimated mean annual progression of SE and AL for children with myopia in previous trials conducted in China aged 9.3 years was estimated at −0.82 D and higher than that reported in the current study (Donovan et al., 2012). Compared to these earlier data, the effect size tends to be higher at nearly 0.48 D with CARE and 0.36 D with CARE S. Adding more real‐world data to the virtual control groups with appropriate validation is possibly required prior to widespread use of such methods.

The strength of this trial is the multi‐centre nature of the trial. This allows for greater confidence in the generalisability of the trial data to the wider population. Furthermore, the low discontinuation rate over the 12‐month period as well as the reported compliance provides confidence in the wearability of these lenses. Additionally, we employed an intent‐to‐treat principle rather than analysing the data on those that completed 12 months in the trial. There are some limitations of the trial. Although the trial was prospective and double‐masked, due to the visibility of the lens features with the myopia control lens designs, masking may not have been possible. However, this is unlikely to have affected the trial outcome as the discontinuation rate was low and compliance was high across all the groups. Additionally, the self‐reporting nature of the subjective assessments conducted in this trial with respect to compliance and wearability are subject to various biases such as recall bias, fatigue with repeated assessments and positive responses. We had not also considered the impact of risk factors such as outdoor time and near work which might be potential confounders. Furthermore, the results are interim and will need to be confirmed with longer duration in lens wear.

In summary, the interim 1‐year results from a multi‐centre clinical trial demonstrate the efficacy of CARE spectacle lenses in slowing myopia. The differences of >0.25 D difference in SE and >0.10 mm difference in AL in comparison to single‐vision spectacle lenses were significant, and additionally, CARE lenses were found to reduce the risk of fast progression.

## CONFLICT OF INTEREST STATEMENT

Arne Ohlendorf, Katharina Rifai, Christina Boeck‐Maier, Siegfried Wahl, Youhua Yang, Yi Zhu and Padmaja Sankaridurg are employees of Carl Zeiss Vision. The other authors have no conflict of interest.

## Supporting information


**Table S1.** Spherical equivalent refractive error and axial length at baseline, 6‐ and 12‐month visit.
