# Supplementary material for: Slowing myopia progression with cylindrical annular refractive elements (CARE) spectacle lenses—Year 1 results from a 2‐year prospective, multi‐centre trial
Source: Acta Ophthalmol. 2024 Nov 15;103(8):929–38. doi: 10.1111/aos.16795 (PMC12604440; doi:10.1111/aos.16795)
Supplement: Supplementary file 1 — Table S1. Spherical equivalent refractive error and axial length at baseline, 6‐ and 12‐month visit. [file AOS-103-929-s001.docx]

Supplementary Table 1: Spherical equivalent refractive error and axial length at baseline, 6- and 12-month visit.

|  | CARE | CARE S | Single Vision | p-value |
| --- | --- | --- | --- | --- |
| Baseline | | | | |
| AL (mm) | 24.34±0.74 | 24.44±0.73 | 24.43±0.73 | 0.870 |
| SE (D) | -2.30±1.06 | -2.23±0.98 | -2.31±1.01 | 0.902 |
| 6 months | | | | |
| AL (mm) | 24.45±0.75 | 24.56±0.74 | 24.61±0.73 | 0.469 |
| SE (D) | -2.40±1.10 | -2.32±1.00 | -2.58±1.02 | 0.278 |
| 12 months | | | | |
| AL (mm) | 24.54±0.74 | 24.68±0.73 | 24.76±0.74 | 0.213 |
| SE (D) | -2.67±1.15 | -2.60±1.06 | -2.95±1.06 | 0.082 |

SE: Spherical equivalent refractive error; AL: Axial Length
